# Advanced Glycation End Products and Activation of Toll-like Receptor-2 and -4 Induced Changes in Aquaporin-3 Expression in Mouse Keratinocytes

**DOI:** 10.3390/ijms24021376

**Published:** 2023-01-10

**Authors:** Yonghong Luo, Rawipan Uaratanawong, Vivek Choudhary, Mary Hardin, Catherine Zhang, Samuel Melnyk, Xunsheng Chen, Wendy B. Bollag

**Affiliations:** 1 Department of Physiology, Medical College of Georgia at Augusta University, Augusta, GA 30912, USA; 2 Department of Medicine (Dermatology), Faculty of Medicine, Vajira Hospital, Navamindradhiraj University, Bangkok 10300, Thailand; 3 Charlie Norwood VA Medical Center, Augusta, GA 30904, USA; 4 Department of Dermatology, Medical College of Georgia, Augusta University, Augusta, GA 30912, USA; 5 Department of Medicine, Medical College of Georgia, Augusta University, Augusta, GA 30912, USA

**Keywords:** toll-like receptor-2 (TLR2), TLR4, advanced glycation end products (AGEs), aquaporin-3 (AQP3), histone deacetylase inhibitor, diabetes, inflammation, keratinocytes, skin

## Abstract

Prolonged inflammation and impaired re-epithelization are major contributing factors to chronic non-healing diabetic wounds; diabetes is also characterized by xerosis. Advanced glycation end products (AGEs), and the activation of toll-like receptors (TLRs), can trigger inflammatory responses. Aquaporin-3 (AQP3) plays essential roles in keratinocyte function and skin wound re-epithelialization/re-generation and hydration. Suberanilohydroxamic acid (SAHA), a histone deacetylase inhibitor, mimics the increased acetylation observed in diabetes. We investigated the effects of TLR2/TLR4 activators and AGEs on keratinocyte AQP3 expression in the presence and absence of SAHA. Primary mouse keratinocytes were treated with or without TLR2 agonist Pam_3_Cys-Ser-(Lys)_4_ (PAM), TLR4 agonist lipopolysaccharide (LPS), or AGEs, with or without SAHA. We found that (1) PAM and LPS significantly upregulated AQP3 protein basally (without SAHA) and PAM downregulated AQP3 protein with SAHA; and (2) AGEs (100 µg/mL) increased AQP3 protein expression basally and decreased AQP3 levels with SAHA. PAM and AGEs produced similar changes in AQP3 expression, suggesting a common pathway or potential crosstalk between TLR2 and AGEs signaling. Our findings suggest that TLR2 activation and AGEs may be beneficial for wound healing and skin hydration under normal conditions via AQP3 upregulation, but that these pathways are likely deleterious in diabetes chronically through decreased AQP3 expression.

## 1. Introduction

Impaired wound healing is one of the major complications of diabetes mellitus. This impairment creates an enormous financial burden on and stress to patients as well as therapeutic challenges for physicians. Unlike acute wounds, wounds with impaired healing do not progress through the four dynamic and overlapping phases necessary for proper healing: hemostasis, inflammation, proliferation, and remodeling. Acute wound healing shows a quick inflammatory response and rapid resolution. In contrast, chronic wound healing is characterized by excessive inflammation and impaired re-epithelization [1,2]. Another characteristic of diabetes is xerosis, i.e., skin dryness resulting from reduced hydration [3]. Aquaporin-3 (AQP3), a water channel that can also transport glycerol and hydrogen peroxide [4,5,6,7,8,9], has been shown to play an important role in regulating proliferation, differentiation and migration of skin epidermal keratinocytes [10,11,12,13,14,15,16,17,18,19,20], as well as skin function, including the water permeability barrier and skin hydration in vivo [20,21]. For example, Verkman and colleagues demonstrated that global AQP3 knockout mice exhibit a skin phenotype of decreased water-holding capacity (hydration) and delayed water permeability barrier recovery, as well as impaired skin wound healing [20,21]. Indeed, in vivo studies have indicated that a diabetes-associated reduction in AQP3 levels may contribute to the skin xerosis observed in diabetes [10,22]. The importance of changes in AQP3 in wound healing in diabetes has also been demonstrated, using a streptozotocin (STZ)-induced diabetic rat model in which impaired re-epithelialization correlated with reduced AQP3 expression during the wound healing process [23]. In addition, siRNA-mediated knockdown of AQP3 in normal human keratinocytes reduces proliferation and migration in wound healing in vitro [10]. Finally, it was recently shown that down-regulation of AQP3 in STZ-induced diabetic mice was not the result of hyperglycemia per se, since one week after STZ injection, serum glucose levels were increased without an accompanying reduction in AQP3 expression. However, a week later (or two weeks after STZ injection), although serum glucose was elevated to a similar level as at one week, AQP3 mRNA and protein levels were decreased [24], suggesting that a diabetic product might need to accumulate to induce AQP3 reduction. We hypothesized that this product might be advanced glycation end products (AGEs).

AGEs are formed through a complicated biochemical process involving non-enzymatic reactions between reducing sugars and free amino groups on proteins, lipids, or nucleic acids. This process is accelerated under chronic hyperglycemic and oxidative stress conditions, which results in AGEs accumulation in high amounts in diabetes [25,26]. In turn, AGEs, through interaction with the receptor for AGEs (RAGE), can activate downstream intracellular signaling pathways that lead to further oxidative stress and production of pro-inflammatory mediators [25,27]. In a diabetic mouse model AGEs have been shown to impair wound healing via delayed infiltration of inflammatory cells, sustained expression of inflammatory mediators, and diminished re-epithelization, and these adverse effects can be inhibited via the use of blockers of RAGE [28,29].

On the other hand, diabetes not only results in increased serum levels of AGEs but has also been found to enhance protein acetylation in various cell types both in vitro and in vivo [30,31,32,33]. This effect is presumed to be mediated by diabetes-related hyperglycemia-induced increases in the generation of the acetyl-CoA required for lysine acetylation. Protein acetylation can also be enhanced by treating cells with the pan-histone deacetylase inhibitor, suberanilohydroxamic acid (SAHA). Therefore, it is possible that SAHA treatment of mouse keratinocytes may mimic some of the effects of diabetes on protein acetylation. Diabetes is also characterized by inflammation, as well as increased serum levels of high-mobility group box 1 (HMGB1) protein [34], an endogenous protein reported to activate toll-like receptors (TLRs), such as TLR2 and TLR4 [34]. TLR2 and TLR4 activation has been demonstrated to both enhance and impair skin wound healing [35]. TLR2 or TLR4 activation during the early healing process improves wound closure, and long-term deficiency of these TLRs delays normal wound healing [1,36,37]. In contrast, in diabetic wounds, the observed extensive expression of TLR2 and TLR4 seems to contribute to increased inflammation and impaired wound closure, while their knockout improves healing [38,39].

The link between AGEs, as well as TLR activation, and AQP3 expression has not been explored. Therefore, we activated TLR2, TLR4, or RAGE with the triacylated synthetic lipopeptide, Pam_3_CSK_4_ (PAM), lipopolysaccharide (LPS) or AGEs, respectively, in the presence or absence of SAHA, to examine AQP3 expression in primary mouse keratinocytes basally and in the presence of SAHA. Treatment with SAHA served to increase the low-level basal expression of AQP3 [40], such that inhibitory effects might be more readily detected. SAHA also enhances protein acetylation, which has been observed in the diabetic setting [30,31,32,33].

## 2. Results

### 2.1. AGE-BSA Used Initially Was Contaminated with Endotoxin

**Figure 1:**
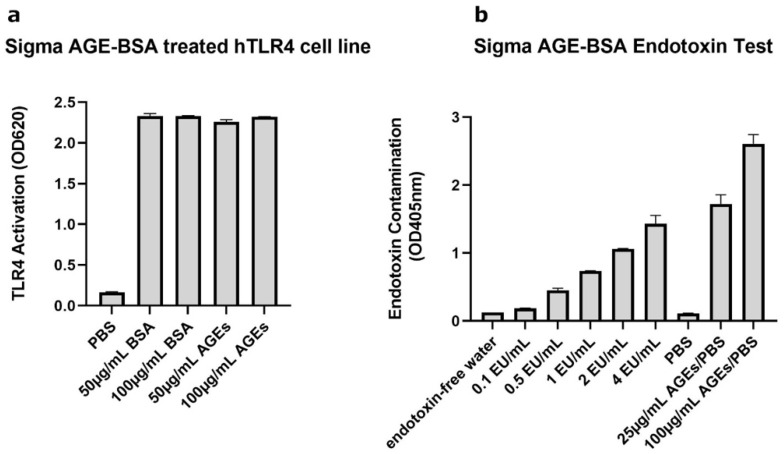
Sigma AGE-BSA Showed Contamination with TLR-Activating Endotoxin. (**a**) The TLR4 reporter cell line HEK-Blue hTLR4 was incubated with Sigma BSA or Sigma AGE-BSA at the indicated concentrations and TLR4 activation measured as a change in absorbance at 620 nm after 24 h. (**b**) Endotoxin contaminating the Sigma AGE-BSA was determined using a Pierce Chromogenic Endotoxin Quant Kit, according to the manufacturer’s directions. Shown are representative experiments performed in duplicate.

Our initial experiments using AGEs obtained from Sigma showed that this reagent reduced the levels of the glycosylated form of AQP3 in mouse and human keratinocytes either in the absence (human) or presence (mouse) of SAHA (Supplementary Figure S1). In human keratinocytes, this reduction was accompanied by a decrease in its function, as measured by glycerol uptake (Supplementary Figure S2). The question then arose as to the mechanism by which the AGEs were affecting AQP3 levels. Reports in the literature suggested that AGEs might serve as endogenous damage-associated molecular patterns (DAMPs) to activate TLR4 [41,42]. To determine whether AGEs activated TLR4, we used a TLR4 reporter cell line and incubated with different concentrations of AGEs or BSA (as a control) obtained from Sigma (St. Louis, MO, USA). As shown in Figure 1a, Sigma AGEs did, in fact, activate TLR4; however, Sigma BSA itself caused as much activation of TLR4 as did the AGEs, suggesting that the Sigma BSA-AGEs (and the BSA from which the AGEs are generated) might be contaminated with a TLR4 activator. In fact, when we measured the amount of endotoxin in the Sigma AGEs using a Pierce^TM^ Chromogenic Endotoxin Quant Kit, the AGEs (at both 25 and 100 µg/mL) showed endotoxin levels greater than the highest concentration tested in the standard curve (4 EU/mL) (Figure 1b). These results confirmed that the Sigma AGEs were contaminated with endotoxin, and it became unclear whether the changes in AQP3 protein expression observed initially were due to the AGEs themselves, to the contaminating endotoxin, or to both. 

### 2.2. TLR2 Activation Downregulated AQP3 mRNA Expression; TLR2/TLR4 Activation Upregulated AQP3 Protein in the Absence of SAHA, and TLR2 Activation Downregulated AQP3 Protein in the Presence of SAHA in Primary Mouse Epidermal Keratinocytes

**Figure 2:**
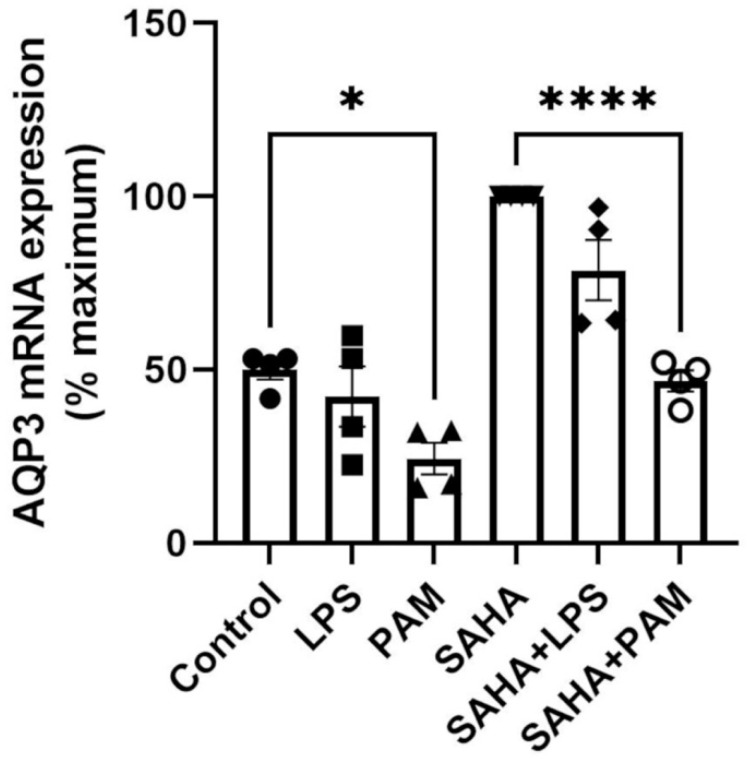
TLR2 Activation Decreased AQP3 mRNA Expression. Mouse keratinocytes were treated with 0 and 2.0 µg/mL LPS or 2.5 µg/mL PAM, in the presence or absence of 1 µM SAHA, for 24 h. mRNA expression of AQP3 was monitored by quantitative RT-PCR. The data are shown as % maximal response and represent the means ± SEM from at least four independent experiments; symbols represent individual experiments. * *p* < 0.05 and **** *p* < 0.0001 versus the control value.

**Figure 3:**
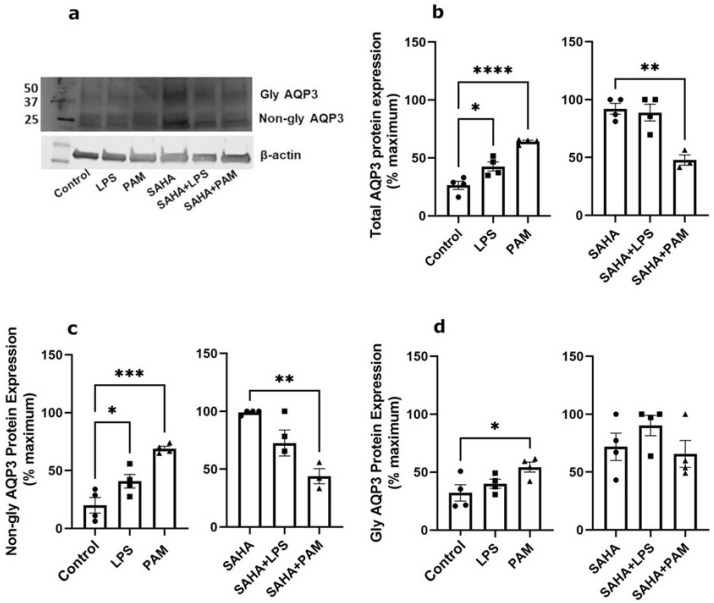
TLR2/TLR4 Activation Increased AQP3 Protein Expression Basally and TLR2 Reduced SAHA-induced AQP3 Levels. Mouse keratinocytes were treated with 0 and 2.0 µg/mL LPS or 2.5 µg/mL PAM, in the presence or absence of 1 µM SAHA, for 24 h. Cells were lysed in 3% boiling SDS lysis buffer and processed for Western analysis. (**a**) Representative Western blot showing APQ3 protein expression. (**b**) Left panel: total AQP3 protein expression in the absence of SAHA. Right panel: total AQP3 protein expression in the presence of SAHA. (**c**) Left panel: non-glycosylated AQP3 protein expression in the absence of SAHA. Right panel: non-glycosylated AQP3 protein expression in the presence of SAHA. (**d**) Left panel: glycosylated AQP3 protein expression in the absence of SAHA. Right panel: glycosylated AQP3 protein expression in the presence of SAHA. The data are expressed as % maximal response among all six groups, with the values in the absence and presence of SAHA analyzed separately and representing the means ± SEM from 3 to 4 independent experiments; symbols represent individual experiments. Note that one data point corresponding to the PAM treatment in the presence of SAHA from the total AQP3 protein expression ((**b**), right panel) and one from the non-glycosylated AQP3 protein expression ((**c**), right panel) were identified as significant outliers (*p* < 0.05) by the GraphPad Outlier Calculator and were removed from the statistical analysis. * *p* < 0.05, ** *p* < 0.01, *** *p* < 0.001, **** *p* < 0.0001 versus the control value.

To test the relative importance of TLR activation in regulating AQP3 expression, keratinocytes were treated with and without a TLR2 or a TLR4 activator in the presence and absence of SAHA. Pam_3_CSK_4_ has been previously shown to increase inflammatory mediator production and NF-κB activation in keratinocytes downstream of TLR2 [43,44]; LPS stimulates keratinocyte inflammatory mediator production downstream of TLR4. TLR2, but not TLR4, activation significantly inhibited AQP3 mRNA expression either in the presence or absence of SAHA (Figure 2). Similarly, TLR2 activation decreased SAHA-induced AQP3 protein expression (Figure 3b, right panel). In contrast, TLR2 and TLR4 activation significantly increased AQP3 protein level basally (Figure 3b, left panel). AQP3 presents as two bands upon Western analysis: an approximately 28 kDa non-glycosylated form and an about 40 kDa glycosylated form [45] (Figure 3a). We, therefore, further analyzed glycosylated and non-glycosylated AQP3 separately. We found that (1) TLR4 activation significantly increased non-glycosylated AQP3 protein expression in the absence of SAHA (Figure 3c, left panel); and (2) TLR2 activation significantly increased both non-glycosylated and glycosylated AQP3 protein expression without SAHA (Figure 3c,d, left panels) but decreased non-glycosylated AQP3 levels in the presence of SAHA (Figure 3c, right panel). Interestingly, we observed increased AQP3 protein levels, despite an inhibitory effect of TLR2 activation on mRNA expression. Since reductions in protein levels tend to lag behind decreases in mRNA expression, particularly if the protein is stable, it seems possible that later time points might be required to observe a decrease in AQP3 levels with PAM treatment.

### 2.3. AGE-BSA from BioVision, Inc. Was Not Contaminated with TLR-Activating Endotoxin

**Figure 4:**
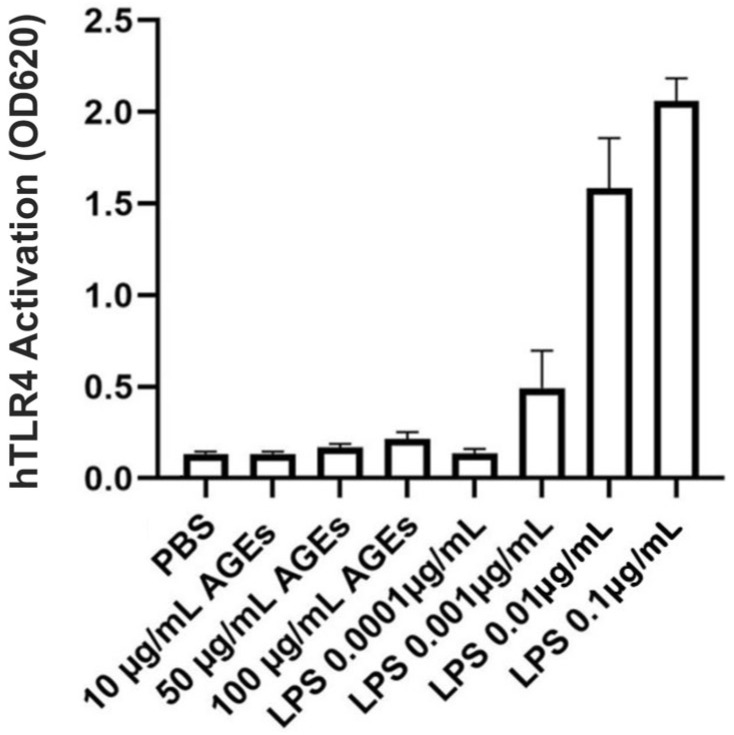
BioVision AGE-BSA Exhibited Little TLR4-Stimulating Activity. HEK-Blue hTLR4 reporter cells were incubated with the indicated concentrations of BioVision AGE-BSA or LPS (as a positive control), and TLR4 activation was measured as a change in absorbance at 620 nm after 24 h. Results represent the means ± SEM of four independent experiments performed in duplicate.

The datasheet provided by BioVision, Inc., (Waltham, MA, USA) for their AGE-BSA indicates endotoxin contamination of less than 0.1 IU/mg protein. To determine whether uncontaminated AGEs can serve as TLR-activating DAMPs, the HEK-Blue TLR4 reporter cell line was incubated with various doses of AGE-BSA from BioVision, Inc. Concentrations of AGEs ranging from 10 to 50 µg/mL showed no activation of TLR4 compared to the phosphate-buffered saline (PBS) control. Even at a concentration of 100 µg/mL, AGEs produced minimal TLR4 activation (Figure 4). Therefore, the AGEs from BioVision, Inc. did not serve as DAMPs to activate TLR4 and did not appear to be contaminated with endotoxin.

### 2.4. AGEs Increased AQP3 Protein in the Absence of SAHA but Reduced AQP3 Protein in Its Presence in Primary Mouse Epidermal Keratinocytes

**Figure 5:**
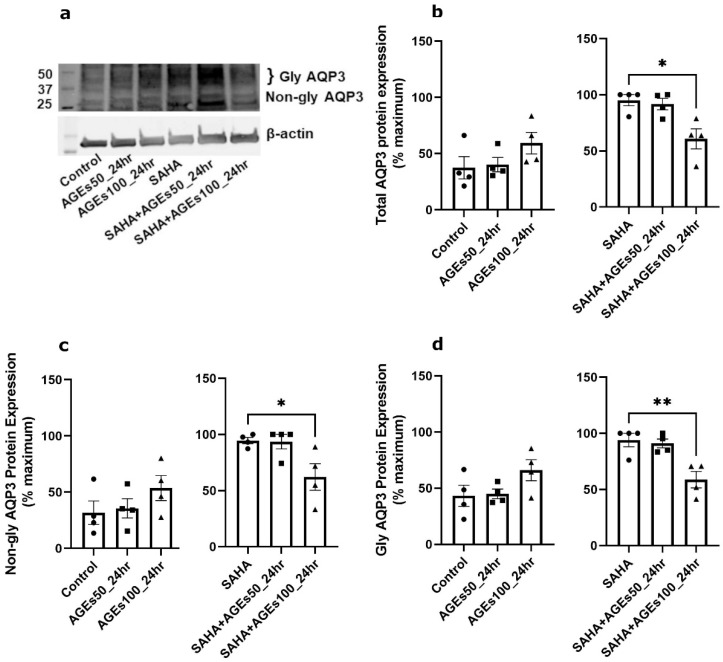
A 24-h Treatment with BioVision AGEs Altered Keratinocyte AQP3 Protein Levels. Western blotting of mouse keratinocytes treated for 24 h with 50 or 100 µg/mL BioVision AGEs. (**a**) Representative Western blot showing AQP3 protein expression. (**b**) Left panel: total AQP3 protein expression in the absence of SAHA. Right panel: total AQP3 protein expression in the presence of SAHA. (**c**) Left panel: non-glycosylated AQP3 protein expression in the absence of SAHA. Right panel: non-glycosylated AQP3 protein expression in the presence of SAHA. (**d**) Left panel: glycosylated AQP3 protein expression in the absence of SAHA. Right panel: glycosylated AQP3 protein expression in the presence of SAHA. The data are analyzed as in Figure 3 and expressed as % maximal response; values represent the means ± SEM from four independent experiments, with symbols representing individual experiments. * *p* < 0.05 and ** *p* < 0.01 versus the control value.

**Figure 6:**
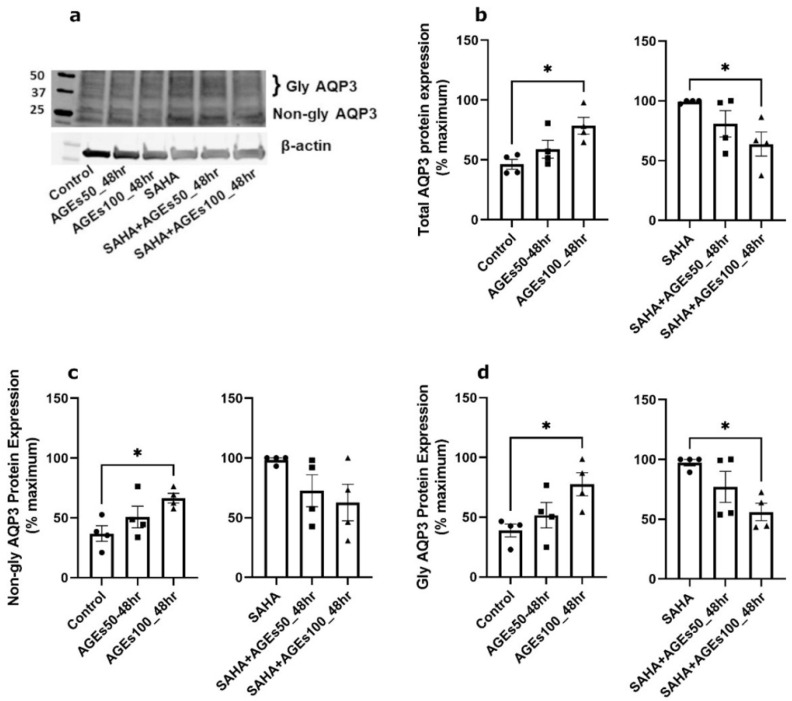
A 48-h Treatment with BioVision AGEs Altered Keratinocyte AQP3 Protein Levels. Western blotting of mouse keratinocytes treated for 48 h with 50 or 100 µg/mL AGEs. (**a**) Representative Western blot showing AQP3 protein expression. (**b**) Left panel: total AQP3 protein expression in the absence of SAHA. Right panel: total AQP3 protein expression in the presence of SAHA. (**c**) Left panel: non-glycosylated AQP3 protein expression in the absence of SAHA. Right panel: non-glycosylated AQP3 protein expression in the presence of SAHA. (**d**) Left panel: glycosylated AQP3 protein expression in the absence of SAHA. Right panel: glycosylated AQP3 protein expression in the presence of SAHA. The data are analyzed as in Figure 3 and expressed as % maximal response; values represent the means ± SEM from four independent experiments, with symbols representing individual experiments. * *p* < 0.05 versus the control value.

**Figure 7:**
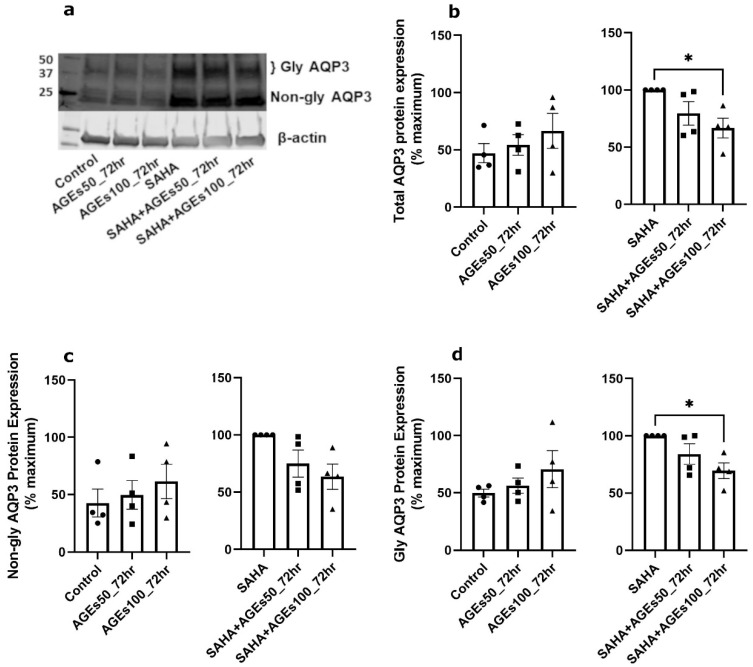
A 72-h Treatment with BioVision AGEs Altered Keratinocyte AQP3 Protein Levels. Western blotting of mouse keratinocytes treated for 72 h with 50 or 100 µg/mL AGEs. (**a**) Representative Western blot showing AQP3 protein expression. (**b**) Left panel: total AQP3 protein expression in the absence of SAHA. Right panel: total AQP3 protein expression in the presence of SAHA. (**c**) Left panel: non-glycosylated AQP3 protein expression in the absence of SAHA. Right panel: non-glycosylated AQP3 protein expression in the presence of SAHA. (**d**) Left panel: glycosylated AQP3 protein expression in the absence of SAHA. Right panel: glycosylated AQP3 protein expression in the presence of SAHA. The data are analyzed as in Figure 3 and expressed as % maximal response; values represent the means ± SEM from four independent experiments, with symbols representing individual experiments. * *p* < 0.05 versus the control value.

We next investigated whether uncontaminated AGEs from BioVision, Inc. would affect AQP3 expression in primary mouse keratinocytes at 24 h (Figure 5), 48 h (Figure 6) and 72 h (Figure 7). Our results revealed that a 24- and 72-h treatment with AGEs at a concentration of 100 µg/mL (AGEs100) tended to increase AQP3 protein levels in the absence of SAHA (Figure 5b and Figure 7b, left panels), although the results did not attain statistical significance. For the 48-h treatment, AGEs100 significantly increased AQP3 protein levels without SAHA (Figure 6b, left panel). In contrast, AGEs100 significantly reduced AQP3 protein expression in the presence of SAHA for each of the 24-, 48-, and 72-h treatments (Figure 5b, Figure 6b and Figure 7b, right panels). We further analyzed glycosylated and non-glycosylated AQP3 separately with or without SAHA. We found that (1) in the absence of SAHA, for the 48-h treatment, AGEs100 significantly increased both non-glycosylated and glycosylated AQP3 protein levels (Figure 6c,d, left panels). For the 24- and 72-h AGEs100 treatment, AQP3 protein expression showed the same tendency as the 48-h AGEs100 treatment even though statistical significance was not achieved (Figure 5c,d and Figure 7c,d, left panels); (2) in the presence of SAHA, AGEs100 significantly decreased both non-glycosylated and glycosylated AQP3 protein (Figure 5c,d, right panels) for the 24-h treatment and significantly reduced glycosylated AQP3 for the 48- and 72-h treatment (Figure 6d and Figure 7d, right panels). Our results suggest that AGEs affected AQP3 protein expression in a dose-dependent manner, with different effects basally versus under conditions of enhanced protein acetylation as has been observed in diabetes [30,31,32,33].

### 2.5. In Primary Mouse Epidermal Keratinocytes, AGEs, but Not SAHA, Increased Cell Number, a Measure of Proliferation

**Figure 8:**
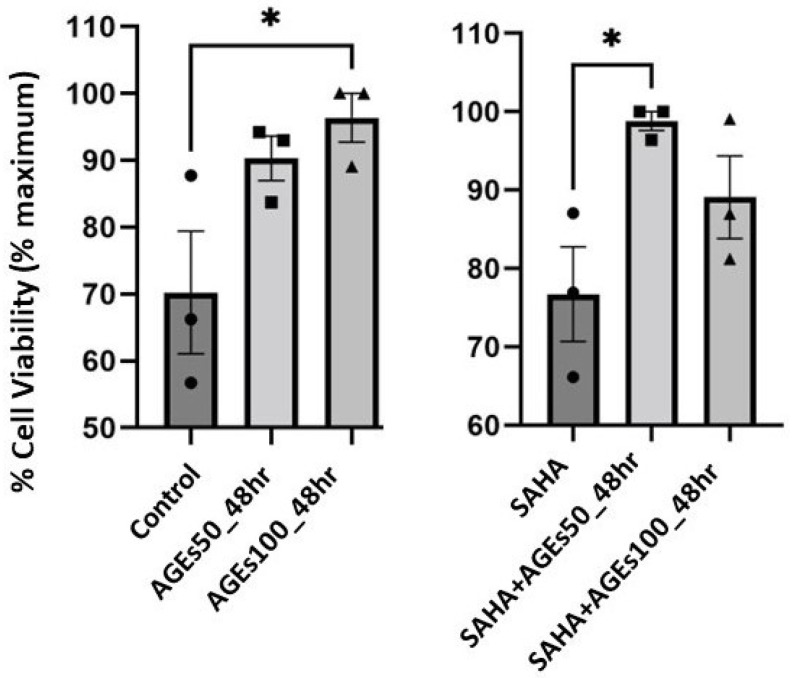
AGEs, not SAHA, IncreasedProliferation in Primary Mouse Keratinocytes. Mouse primary keratinocytes were treated with BioVision AGEs in the presence of absence of 1 µM SAHA for 48 h. Cell proliferation was measured using MTT assays. The data are expressed as % maximal response and represent the means ± SEM from three independent experiments; symbols represent individual experiments. * *p* < 0.05 versus the control value.

We also determined the effect of a 48-h treatment with BioVision AGEs (50 and 100 µg/mL) on cell numbers in primary mouse keratinocytes treated with or without 1 µM SAHA, as measured using 3-(4,5-dimethylthiazol-2-yl)-2,5-diphenyltetrazolium bromide (MTT) assays. The MTT assay provides information about metabolic activity; however, since this activity is proportional to the number of viable cells, the intensity of the colored product also yields data about cell number and thus cell proliferation [46]. In the absence of SAHA, AGEs at a concentration of 100 µg/mL significantly enhanced proliferation. In the presence of SAHA, AGEs at a concentration of 50 µg/mL but not 100 µg/mL, increased proliferation. Our data also showed no effect of 1 µM SAHA on mouse keratinocyte proliferation (Figure 8).

## 3. Discussion

AQP3 is an important protein that regulates keratinocyte proliferation, differentiation, and migration, as well as skin hydration and water permeability repair in vivo, and dysregulation of AQP3 leads to impaired wound healing [10,11,12,13,14,15,16,17,18,19,20] and xerosis [10,22]. The major findings of this study are that TLR2 activation and AGEs (100 µg/mL) upregulated AQP3 protein levels in the absence of SAHA, but TLR2 activation and AGEs (100 µg/mL) downregulated AQP3 protein levels in the presence of SAHA in primary mouse keratinocytes. Interestingly, both TLR2 activation and AGEs (100 µg/mL) had similar effects on AQP3 protein expression, suggesting a common pathway or potential crosstalk between TLR2 and RAGE. The principal mechanism(s) of how AQP3 is regulated by TLR2 activation and AGEs is (are) currently unclear. Nonetheless, both TLR2 activation and AGEs can activate the NF-κB pathway, leading to release of pro-inflammatory cytokines [28,35,47]. A secondary finding of potential significance is our data demonstrating that AGEs that were not contaminated with endotoxin did not activate TLR4, contrary to some reports in the literature [48,49].

In the absence of SAHA, TLR2 stimulation or AGEs (100 µg/mL) treatment of mouse keratinocytes enhanced AQP3 protein expression. The upregulation of AQP3 should enhance the transport function of AQP3, a water, glycerol, and hydrogen peroxide channel, as shown by changes in glycerol transport with endotoxin-contaminated AGEs and SAHA in Supplementary Figure S2. Through glycerol uptake and subsequent cell swelling, AQP3 can promote NLRP3 inflammasome activation and pro-inflammatory cytokine production [50]. The activation of the NLRP3 inflammasome has been shown to enhance skin wound healing by elevating pro-inflammatory cytokine production in the wound site [51,52]. Additionally, increased AQP3 in keratinocytes should enhance their proliferation, differentiation, and migration to contribute to wound healing, as has been shown in various studies in human and mouse keratinocytes (reviewed in [13,53]). Indeed, AGEs (100 µg/mL) enhanced the basal proliferation of primary mouse keratinocytes. This result is consistent with the finding that AGEs (100 µg/mL) enhance wound healing in a human corneal epithelial cell line via RAGE activation [54], and homozygous RAGE null mice exhibit delayed re-epithelialization in mouse corneal wounds [55]. Our results therefore suggest that TLR2 activation and AGEs may have a beneficial effect on acute wound healing in part through upregulation of AQP3 under basal conditions.

On the other hand, in the presence of SAHA, AGEs (100 µg/mL) or the TLR2 agonist PAM reduced AQP3 protein levels in mouse keratinocytes. As SAHA increases the low basal AQP3 protein expression [40], treatment with this agent allows a greater ability to detect decreases in AQP3 levels. SAHA-mediated HDAC inhibition may also mirror the protein hyperacetylation observed in diabetes, although at this point it is unclear whether or not SAHA affects the acetylation of the same or different proteins as in diabetes. In addition, it is likely that HDAC inhibitors have distinctive effects on different cell types. Thus, some studies report that HDAC inhibitors promote β-cell development and proliferation and might potentially therefore be a novel treatment for diabetes [56,57,58], whereas our data indicated no effect of 1 µM SAHA on mouse epidermal keratinocyte proliferation (Figure 8). The ability of HDAC inhibitors to increase β-cell numbers might suggest their potential therapeutic use in diabetes, despite the increased protein acetylation observed in other tissues in individuals with this disease [30,31,32,33]. In addition, HDAC inhibitors exhibit anti-inflammatory effects [59,60] that might be useful in treating diseases like diabetes.

There are limitations to our study. Thus, our study was restricted to in vitro experiments examining cell growth only, and not migration. Another limitation of our study is the fact that we have not defined the mechanisms by which downregulation of AQP3 might affect wound healing in diabetes. However, based on the literature, we can speculate that decreased AQP3 might inhibit reswelling-induced activation of NLRP3-inflammasome to reduce production of pro-inflammatory mediators [50] and hinder the orderly progression of inflammation at the wound site, contributing to delayed wound healing [51,52]. Indeed, multiple lines of evidence support the idea that chronic increases in AGEs in diabetes impair skin wound healing, a function of proliferation and migration, in vivo [61]. Thus, our data support the hypothesis that accumulation of AGEs may downregulate AQP3 expression in diabetes and may explain the findings of Ikarashi et al. [24] that an elevation in blood glucose levels was not sufficient to decrease AQP3 expression but required time to develop (to allow AGEs to form) in diabetic mice. Indeed, AQP3 knockout mice show impaired wound healing accompanied by reduced proliferating keratinocytes, and siRNA-induced AQP3 knockdown slows keratinocyte migration [10]. Our data thus suggest that TLR2 activation and/or AGEs formation in diabetes may be deleterious for wound healing, in part via downregulation of AQP3 protein in this condition.

In this study, we also analyzed the effects of AGEs on glycosylated and non-glycosylated AQP3 separately. Both glycosylated and non-glycosylated AQP3 were upregulated by AGEs in the absence of SAHA but downregulated in the presence of this HDAC inhibitor. The exact role of glycosylation in regulating AQP3 function is unclear but by analogy with AQP2 [62], glycosylation may regulate plasma membrane localization of AQP3. Therefore, this effect of AGEs may impair AQP3’s membrane localization and transport function, although further studies are needed to examine this idea.

In conclusion, our study revealed that both TLR2 activation and AGEs treatment (100 µg/mL) of mouse keratinocytes promoted AQP3 protein expression basally, and elevated AQP3 protein should be protective in wound healing. However, with SAHA-mediated HDAC inhibition, TLR2 activation and AGEs decreased AQP3 protein, which is likely detrimental to wound healing, based on the important role of AQP3 in regulating keratinocyte proliferation and migration [10,11,12,13,14,15,16,17,18,19,20]. Our data suggest a possible role for AQP3 in connecting the inflammatory and proliferative phases of wound healing. Further, we speculate that AGEs-induced activation of RAGE acutely following injury may serve to promote wound healing, at least in part through effects on basal AQP3 levels. However, with prolonged elevations in AGEs levels and RAGE activation, as well as other changes accompanying diabetes (e.g., inflammation and enhanced protein acetylation), AGEs instead down-regulate AQP3 to impair skin wound healing. These results suggest that further investigation is warranted to determine the mechanisms by which AQP3 is regulated to exert both beneficial and harmful effects in complex wound healing processes.

## 4. Materials and Methods

### 4.1. Cell Culture and Treatment

Mouse keratinocytes were isolated from the skin of newborn ICR CD1 mice and cultured as described previously [63], according to protocols approved by the Institutional Care and Use Committees of Augusta University (Protocol #2017-0915) and the Charlie Norwood VA Medical Center (Protocol #22-04-135). Keratinocytes were treated with 0, 2.0 µg/mL LPS (Sigma-Aldrich, St. Louis, MO, USA), or 2.5 µg/mL Pam_3_CSK_4_ (EMD Millipore Corporation, Burlington, MA, USA) in the presence or absence of 1 µM SAHA (Sigma-Aldrich, St. Louis, MO, USA) at 37 °C with 5% CO_2_ for 24 h; or with 0, 50, or 100 µg/mL AGE-BSA (Sigma-Aldrich or BioVision Incorporated, Milpitas, CA, USA), in the presence or absence of 1 or 2 µM SAHA (based on our previous studies [40]) at 37 °C with 5% CO_2_ for 24, 48, and 72 h. Doses of AGEs were selected based on previous literature [54,64,65,66]. Cells were then harvested for RT-qPCR or Western analysis. Cells treated with SAHA were analyzed separately, to determine whether TLR2/TLR4 activation or AGEs altered AQP3 expression and/or function under basal conditions and settings analogous to diabetes (with enhanced protein acetylation).

### 4.2. Quantitative RT-PCR

Cells were harvested and RNA isolated using PureLink™ RNA Mini Kits (ThermoFisher Scientific, Waltham, MA, USA). Total RNA was reverse transcribed to cDNA using High-Capacity cDNA Reverse Transcription Kits (ThermoFisher Scientific, St. Louis, MO, USA). Gene expression was analyzed by quantitative RT-PCR using Taqman primer-probe sets purchased from ThermoFisher Scientific according to the supplier’s instructions and the delta-delta Ct method with GAPDH as the housekeeping gene, as described previously [40].

### 4.3. Western Blotting

Western analysis was performed as described previously [40], using a primary anti-AQP3 antibody obtained from Novus Biologicals (Littleton, CO, USA) and secondary IRDye-conjugated antibody purchased from LI-COR Biosciences (Lincoln, NE, USA). Immunoreactive bands were visualized using a LI-COR Odyssey infrared imager and quantified using the LI-COR software. AQP3 levels were normalized to β-actin levels, using an antibody purchased from Sigma-Aldrich (St. Louis, MO, USA), and expressed as the percent maximal response within each experiment and in the presence or absence of SAHA, with results representing the means ± SEM of at least three separate experiments. AQP3 bands were identified in part based on molecular weight and in part on our previous results using AQP3 knockout cells with and without re-expression of AQP3 [16] and up- and down-regulation of the protein [40].

### 4.4. BSA-AGEs Contamination Test

Contamination of Sigma’s BSA-AGEs with TLR4-activated endotoxin was determined using two assays. Initially, a TLR4 reporter cell line was used to monitor TLR4 activation. The HEK-Blue hTLR4 cells (InvivoGen, San Diego, CA, USA) are HEK293 cells engineered to express human TLR4, along with the TLR4 accessory proteins/co-receptors CD14 and MD-2, as well as a secreted embryonic alkaline phosphatase (SEAP) under the control of an interleukin-12 p40 minimal promoter with multiple AP-1 and NF-kB consensus sequences. Thus, activation of TLR4 results in AP-1- and NF-κB-mediated transcription of SEAP, the activity of which can then be measured using the chromogenic substrate in HEK-Blue detection medium. Endotoxin contamination was also measured directly using a Pierce^TM^ Chromogenic Endotoxin Quant Kit (ThermoFisher Scientific), according to the manufacturer’s instructions.

### 4.5. MTT Assay

MTT assays were performed using Roche’s Cell Proliferation Kit I according to the manufacturer’s instructions. Initially, experiments were performed to determine the initial plating density necessary to ensure that the cells were still proliferating, i.e., they had not achieved confluence, at the time of assay completion. Mouse keratinocytes plated at the determined density in 96-well plates were then treated with 0, 50, or 100 µg/mL AGE-BSA (BioVision Incorporated, CA, USA), in the presence or absence of 1 µM SAHA and incubated at 37 °C in 5% CO_2_ for 48 h. Subsequently, MTT (3-[4,5-dimethylthiazol-2-yl]-2,5-diphenyltetrazolium bromide) obtained from Roche Diagnostics GmbH (Mannheim, Germany) was added to each well at a final concentration of 0.5 mg/mL. Cells were incubated for 4 h at 37 °C in 5% CO_2_ to allow purple formazan crystals to form in metabolically active cells. An amount of 100 µL of solubilization buffer was then added into each well and incubated overnight at 37 °C with 5% CO_2_ to dissolve the formazan crystals. Absorbance at 550 nm was measured using a Gen5 96-well plate reader (BioTek Instruments, Winooski, VT, USA).

### 4.6. Statistical Analysis

Results represent the means ± SEM of 3-6 separate experiments and are expressed either as a fold over control or as the % maximal response. Group mean values were compared using one-way analysis of variance (ANOVA) with Tukey tests. All statistical analyses were performed using GraphPad software (San Diego, CA, USA).

## Data Availability

The data are provided within the figures in the manuscript and Supplementary Materials. The raw data underlying these figures will be provided upon reasonable request.

## Supplementary Materials

The following supporting information can be downloaded at: https://www.mdpi.com/article/10.3390/ijms24021376/s1.
