# Supplementary material for: Advanced Glycation End Products and Activation of Toll-like Receptor-2 and -4 Induced Changes in Aquaporin-3 Expression in Mouse Keratinocytes
Source: Int J Mol Sci. 2023 Jan 10;24(2):1376. doi: 10.3390/ijms24021376 (PMC9864132; doi:10.3390/ijms24021376)
Supplement: Supplementary file 1 [file ijms-24-01376-s001.zip › ijms-1822470-supplementary.pdf]

Supplementary Materials

for

Advanced Glycation End Products and Activation of Toll-like Receptor-2 and -4 Induced Changes in Aquaporin-3 Expression in Mouse Keratinocytes

by

Yonghong Luo, Rawipan Uaratanawong, Vivek Choudhary, Mary Hardin, Catherine Zhang, Xunsheng Chen and Wendy B. Bollag

Supplementary Methods

Radiolabeled Glycerol Uptake Assays

Glycerol uptake was determined as previously described [40], after incubating cells with 1 μCi/mL [^{3}H]glycerol for 5 minutes and washing extensively with ice-cold PBS to remove extracellular glycerol. Cells were solubilized with NaOH and radioactivity measured as above. Protein concentration was determined and used to normalize radioactivity also as described [40].

Human Keratinocyte Culture and Treatment

Normal (neonatal) human keratinocytes were obtained from Lonza (Basel, Switzerland) and cultured according to the supplier's instructions. Keratinocytes were treated with 0, 50 or 100 μg/mL AGEs, in the presence or absence of 1 μM SAHA at 70-80% confluence for 24 hours prior to Western analysis of AQP3 protein expression.

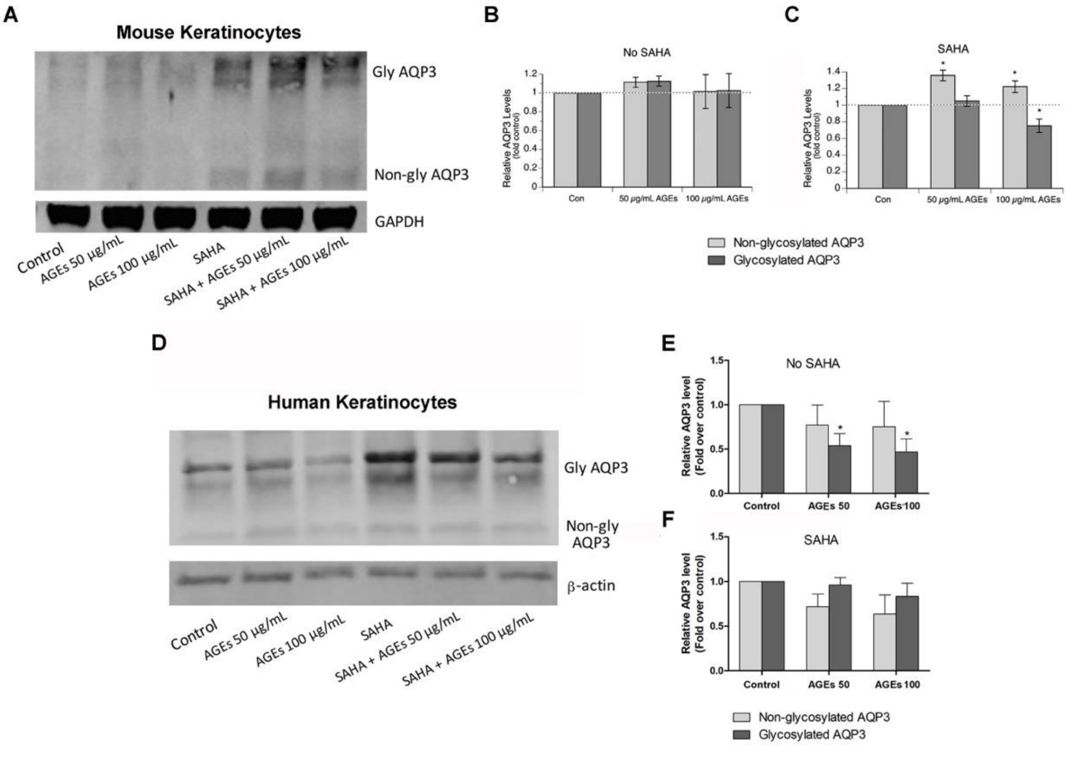

Figure S1. Sigma AGEs decreased glycosylated AQP3 levels in mouse and human keratinocytes. (A-C) Mouse keratinocytes or **(D-F)** human keratinocytes were treated as indicated with or without AGEs in the presence or absence of SAHA for 24 hours. Cells were lysed in warm SDS lysis buffer and processed for Western analysis. **(A)** and **(D)** Representative blots are shown. **(C, D, E** and **F)** Cumulative results from 4 separate experiments are shown as the means ± SEM of the appropriate control with or without SAHA exposure; *p<0.05 versus the control value. Basal AQP3 levels are more readily detected in human keratinocytes, which may allow an effect of 100 μg/mL AGEs to decrease AQP3 levels to be observed. On the other hand, although AGEs tended to reduce AQP3 protein expression in SAHA-treated human keratinocytes, the values did not achieve statistical significance. Nevertheless, the mouse and human keratinocytes respond slightly differently for reasons that are as yet unclear.

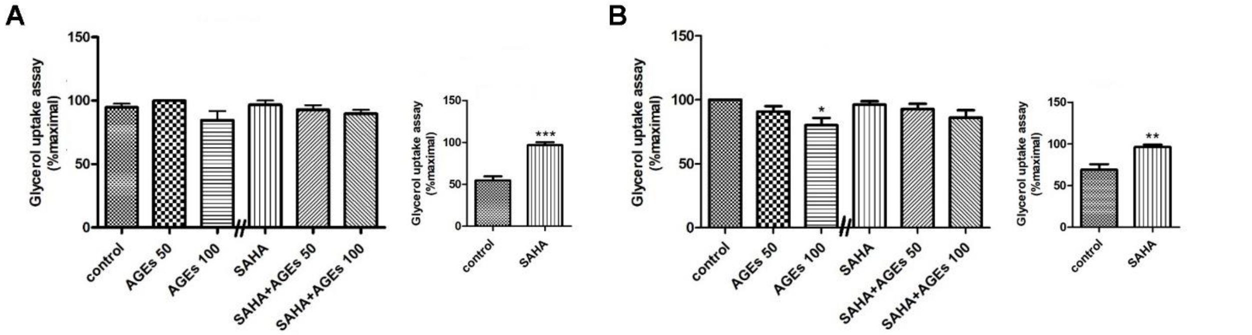

Figure S2. Sigma AGEs reduced glycerol uptake in human keratinocytes. (A) Mouse keratinocytes and **(B)** normal human keratinocytes were treated with or without AGEs, 50 or 100 μg/mL, in the presence or absence of 1 μM SAHA, for 24 hours. [^{3}H]Glycerol (1 μCi/mL) was added for 5 minutes and reactions terminated as described above; scintillation counting was performed and protein used to normalize values. The data are shown as %maximal activity and represent the means ± SEM from 4 independent experiments; *p<0.05, **p<0.01 and ***p<0.001 versus the control.
